# Physical Activity in German Adolescents Measured by Accelerometry and Activity Diary: Introducing a Comprehensive Approach for Data Management and Preliminary Results

**DOI:** 10.1371/journal.pone.0065192

**Published:** 2013-06-04

**Authors:** Rebecca Pfitzner, Lukas Gorzelniak, Joachim Heinrich, Andrea von Berg, Claudia Klümper, Carl P. Bauer, Sibylle Koletzko, Dietrich Berdel, Alexander Horsch, Holger Schulz, for the GINIplus Study Group

**Affiliations:** 1 Institute of Epidemiology I, Helmholtz Zentrum München, German Research Center for Environmental Health, Neuherberg, Germany; 2 Institut für Medizinische Statistik und Epidemiologie, Technische Universität München, Munich, Germany; 3 Department of Pediatrics, Marien-Hospital Wesel, Wesel, Germany; 4 IUF-Leibniz Research Institute for Environmental Medicine at the Heinrich-Heine University of Düsseldorf, Düsseldorf, Germany; 5 Department of Pediatrics, Technische Universität München, Munich, Germany; 6 Dr von Hauner Children’s Hospital, Ludwig-Maximilians-University of Munich, Munich, Germany; Pulmonary Research Institute at LungClinic Grosshansdorf, United States of America

## Abstract

**Introduction:**

Surveillance of physical activity (PA) is increasingly based on accelerometry. However, data management guidelines are lacking. We propose an approach for combining accelerometry and diary based PA information for assessment of PA in adolescents and provide an example of this approach using data from German adolescents.

**Methods:**

The 15-year-old participants comprised a subsample the GINIplus birth cohort (n = 328, 42.4% male). Data on PA was obtained from hip-worn accelerometers (ActiGraph GT3X) for seven consecutive days, combined with a prospective activity diary. Major aspects of data management were validity of wear time, handling of non-wear time and diary comments. After data cleaning, PA and percentage of adolescents meeting the recommendations for moderate-to-vigorous activity (MVPA) per day were determined.

**Results:**

From the 2224 recorded days 493 days (25%) were invalid, mainly due to uncertainties relating to non-wear time (322 days). Ultimately, 269 of 328 subjects (82%) with valid data for at least three weekdays and one weekend day were included in the analysis. Mean MVPA per day was 39.1 minutes (SD ±25.0), with boys being more active than girls (41.8±21.5 minutes vs. 37.1±27.8 minutes, p<0.001). Accordingly, 24.7% of boys and 17.2% of girls (p<0.01) met the WHO recommendations for PA. School sport accounted for only 6% of weekly MVPA. In fact, most MVPA was performed during leisure time, with the majority of adolescents engaging in ball sports (25.4%) and endurance sports (19.7%). Girls also frequently reported dancing and gymnastics (23%).

**Conclusion:**

For assessment of PA in adolescents, collecting both accelerometry and diary-based information is recommended. The diary is vital for the identification of invalid data and non-compliant participants. Preliminary results suggest that four out of five German adolescents do not meet WHO recommendations for PA and that school sport contributes only little to MVPA.

## Introduction

The importance of physical activity (PA) is well documented for several health outcomes. Being physically active is considered to have protective effects on adiposity, mental, cardiometabolic as well as musculoskeletal health [1], [2]. To benefit from these positive effects and to prevent the development of diseases a daily moderate-to-vigorous physical activity (MVPA) of at least 60 minutes per day in adolescents is recommended by the WHO [3]. The interest in individuals at young age is particularly important because acquired behaviour patterns sustain into adulthood [4]. Active adolescents have both a higher chance to be active as an adult and to remain healthy until old age. Inactivity is not only a global risk factor for health [5], [6], but also causes high costs for the health care system [7]–[10]. Therefore, inactivity has become a major public health concern. For this reason, a major goal is to promote PA already during childhood, for both an improved health status and a decrease in social economic cost.

However, studies conducted throughout the world reveal that adolescents are insufficiently active and that activity decreases with increasing age during childhood [11], [12]. According to the WHO study on “Health Behaviour in School-aged Children” (HBSC) and the “German Health Interview and Examination Survey” (KIGGS), less than 25% of the adolescents engage in the recommended daily MVPA [13]–[15]. Results of both studies are based on self-reported data, which are regarded to overestimate PA [16]. The HELENA study was conducted to objectively measure PA in 2,200, 12.5 to 17.5 year old adolescents in different European countries [17]. Among European boys, 56.8% met the PA recommendations of the WHO, whereas only 27.5% of the girls were sufficiently active. Since stratified analyses by country were not conducted data of objectively measured PA from adolescents are limited in Europe and missing in Germany.

The application of accelerometers to measure PA has more than doubled in the last years. In comparison with subjective methods, accelerometry is regarded to be more precise in measurement but more complex in application and interpretation. As a compromise between validity and applicability, accelerometers have proven suitable in large population based studies. Guidelines have been published, providing overall recommendations for methodological aspects such as the measuring schedule, placement and distribution of the acceleration monitors [18]. However, the methodological process in data preparation is still inconsistent between studies and standardization of the methodological processing is long-needed [19]. Several studies recommend the additional use of activity diaries to collect information about daily routines, specific activities, and non-wear time of accelerometers and, thus, provide the option for improved data management [20], [21]. Activity diaries also provide the opportunity to address specific aspects of interest, such as contribution of school sport to overall PA. Having more detailed standards for data management is mandatory in the effort to improve comparability of generated results from different studies.

Using accelerometers and activity diaries in a German cohort of adolescents (GINIplus, [22]–[24]), the aim of the present study was twofold: First, to introduce a comprehensive approach based on recent recommendations and provide detailed information about cleaning of recorded accelerometry data and handling of information derived from an activity diary applied in German adolescents. Second, to provide first results on objectively measured PA in German adolescents and to give an overview of the information derived from the activity diary.

## Methods

### Ethics Statement

The GINIplus study was approved by the local Ethics Committees, the Bavarian General Medical Council (Bayerische Landesärztekammer, Munich, Germany) for the study place Munich and the Medical Council of North-Rhine-Westphalia (Ärztekammer Nordrhein, Düsseldorf, Germany) for Wesel. The approvement of the Ethics Committees includes the written consent procedure. Written informed consent was obtained from the parents or the legal guardian of all participating adolescents.

### Study Population

The participants were comprised of the 15-years follow-up of the “German Infant Nutrition Intervention Programme” (GINIplus) study. The cohort and recruitment of the participants has been described in detail previously [22]–[24]. In brief, GINIplus is a longitudinal study of a German birth cohort born between September 1995 and June 1998 in the cities of Munich (urban, south-east of Germany) and Wesel (rural, north-west of Germany). At baseline, 5,991 participants (2,949 in Munich, 3,042 in Wesel) were recruited. Of these subjects, 3,317 adolescents (55%, 1,730 from Munich and 1,587 from Wesel) are supposed to participate in the ongoing 15-years follow-up.

**Figure 1:**
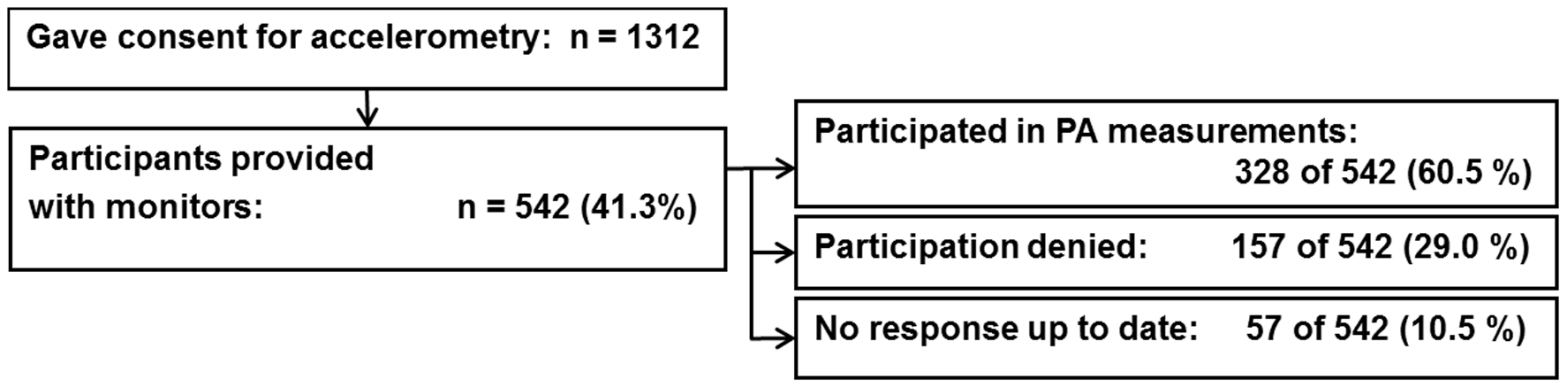
Response rate to accelerometry in the ongoing GINIplus study (date: June 2012).

At the time of data evaluation for the present study (June 2012), 1,312 adolescents had given consent to conduct PA measurements by accelerometry. 542 of the 1,312 adolescents (41.3%) had been contacted and asked to monitor PA. Of those contacted, 328 (60.5%) were willing to participate in PA measurements and returned monitors with recorded data up to date (Figure 1). Although consent was given, 157 of 542 adolescents (29.0%) denied participation after receiving monitors. According to comments in the diary, or after phone call, most of the reasons for denial concerned the size and appearance of the monitors. In 57 of 542 cases (10.5%) no response was obtained up to date.

### Accelerometry

To assess the PA of the study sample, two triaxial accelerometers (ActiGraph GT3X, Pensacola, Florida) were used, one worn at the hip and another one at the ankle. One monitor was attached to an elastic belt at the hip on the side of the dominant hand. Overnight the monitor was moved from hip to the wrist on the non-dominant hand side to measure the sleep quality using an established algorithm from Sadeh et al. [25], [26]. The other monitor was mounted at the dominant ankle. In the present study, only information of the hip-worn accelerometer was analysed, concordant with existing recommendations [18]. The evaluation of the sleep quality will be presented elsewhere. ActiGraph accelerometers were chosen for reasons of application in and comparability to other studies. ActiLife software was used for initialization of accelerometers (version 5.5.5, firmware 4.4.0). The sampling rate was set to 30 Hz and the measured accelerations stored at 1 Hz after conversion into proprietary “activity count units” summed over a second-by-second time interval. Data filtering was set to default (‘normal’) recommended by ActiGraph. Activity counts of all three axes (vertical, horizontal and mediolateral), the inclinometer signal, and number of steps were measured over one week, starting in the morning of the first school day after subjects received the monitors (see below).

### Approach of Participants

Participants were typically contacted around their 15^th^ birthday. Although considered as best practice [18], participants were not contacted and instructed face-to-face, due to the large sample size and regional distance from the study centre. Instead a mailing bag was send to participants, as suggested by Matthews et al. [18]. The bag included a personalized cover letter, a brief instruction sheet, the pre-printed diary journal, two monitors with an identification label for hip and foot at the upside including mounting strips, and a stamped, self-addressed envelope for returning monitors after one week of PA measurement. Participants were asked to start measurements at the morning of the first school day after receiving the monitors. After monitors were sent back and data was analyzed, participants received a result letter, containing diagrams of their daily physical activity intensity and the sleep quality (Figure S1). It served as an incentive for their participation. The result letter contained recommendations to increase or maintain the current physical activity level according to the proven recommendation of 60 minutes MVPA per day [3].

### Activity Diary

Adolescents were instructed to record the course of their days in an activity diary using a detailed schedule (Figure S2). First, anthropometric information such as weight and height, participant’s handedness and hand side of wearing the monitor was recorded. Second, the following events were reported per day and illustrated in a sample how to fill in the diary: the time and reason of removing one or both monitors (non-wear time), e.g. for showering or swimming; waking up; travel to and from school; kind of transportation (e.g. by bike or bus); time of being in school; school sports; end of school; and (if any) sport activities, including type of sport. Furthermore, information about time of going to bed and time getting up from bed was recorded. It was equivalent to the time of changing the monitor from hip to wrist in the evening, and vice versa in the morning. This information was used to distinguish between self-reported day and night activities. Participants were also asked to rate their sleep quality and well-being during the day using a proven instrument [27]. A note at the end of the 7^th^ recording day reminded the participant to stop the measurement and immediately return monitors and diary to the study centre (Figure S2).

### Data Management and Quality Control

**Figure 2:**
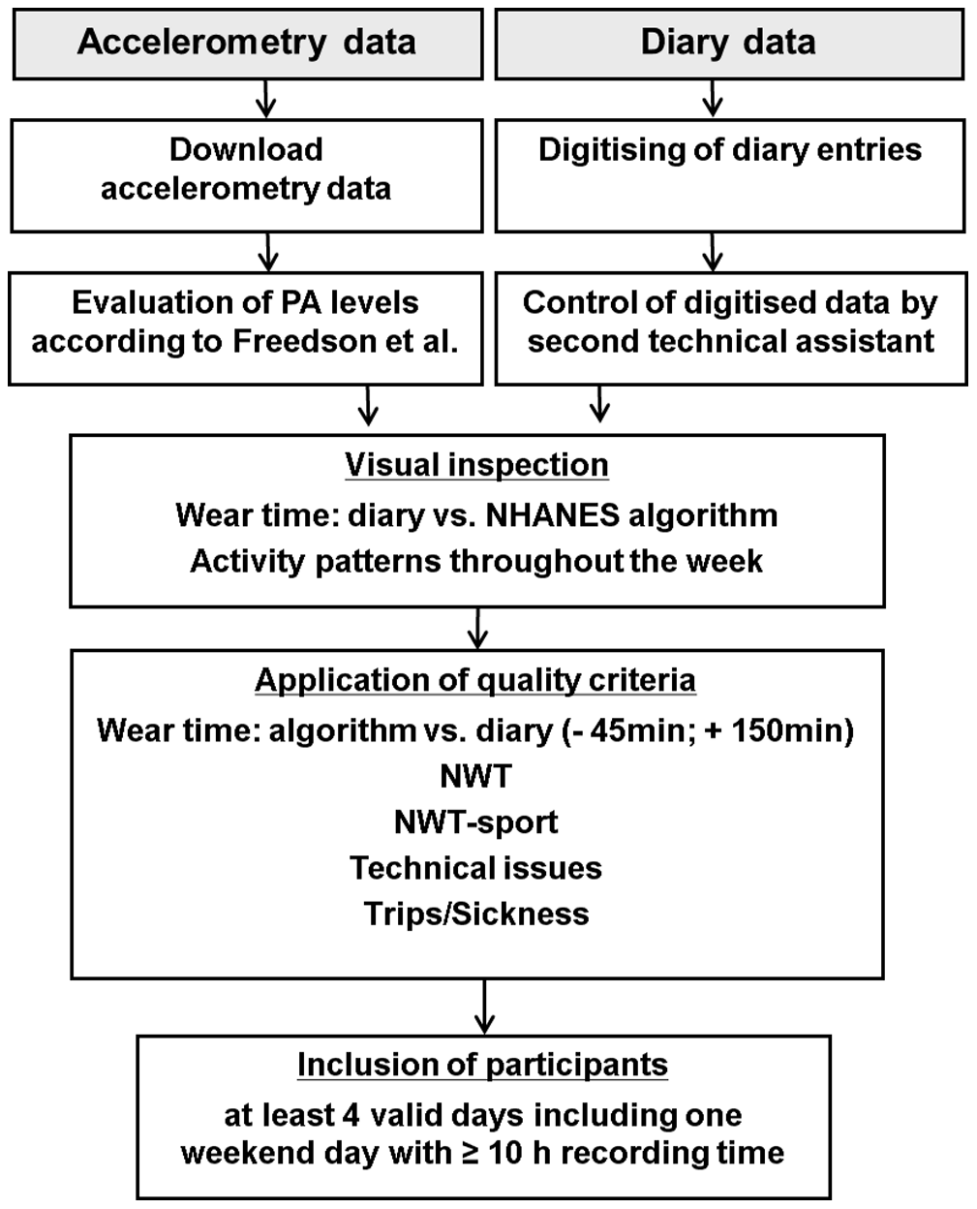
Process of data management and criteria of quality control in the ongoing GINIplus study. (NWT: non-wear time, NWT-sport: non-wear time during sport activities, Freedson et al. 2005 [29], NHANES algorithm for detecting NWT [33]).

This process included the following activities: download and evaluation of the PA data; digitisation of diary information; validation of wear time and handling of sensor non-wear time (NWT); and identification of recording errors. The PA data were initially checked by visual inspection and then subjected to semiautomatic standards for quality control to identify invalid days or days with insufficient data (Figure 2, [28]).

#### Management of diary and accelerometer data

Diary information was digitised using a 7-day template and a specific coding for events such as sickness, trips, type of sport performed, and NWT. Data entries were reviewed by a second study assistant to avoid transcription errors.

The PA data recorded were downloaded using the ActiLife Software for both 1 second and 60 seconds epoch lengths. The present data analysis used data from the hip-worn monitor and focused on the established 60 seconds epoch length. Activity counts of the vertical axis were assigned to the four intensity levels, sedentary, light, moderate, and vigorous physical activity using frequently applied cut points published by Freedson et al. [29]. Although the data was recorded on three axes, uniaxial cut points were applied due to the scarcity of currently available, valid triaxial ActiGraph cut points for adolescents. The comparability of the vertical axis of the GT3X with previous ActiGraph monitors has been proven [30], [31]. Further, based on results from McClain et al. [32] Freedson’s cut points are insensitive to different epoch lengths with respect to times spent in MVPA, other than Mattocks’ or Treuth’s cut points which could have been an alternative. The minutes spent in each of the four intensity levels were cumulated for each measuring day of the 7 day measuring period, e.g. Monday (MO) to Sunday (SU, Figure S3).

#### Validity of wear time

This process aimed to identify discrepancies in wear time according to accelerometer data and diary information. An established algorithm of the National Health and Nutrition Examination Survey (NHANES) was used for this purpose [33]. The algorithm aims to identify NWT periods and is based on the vertical axis counts of the hip worn monitor. Tests for periods with consecutive zero counts of at least 60 minutes including less than two consecutive intervals with counts less than or equal to 100 are performed. Graphic representations of the wear time according to the NHANES algorithm and information about wear time stated in the activity diary were visually inspected for each participant (Figure S3). In most cases diary and algorithm agree upon wear time and NWT, so that NWT could reliably be excluded from data analysis. In the event of large discrepancies between algorithm and diary entry valid evaluation of PA is not guaranteed and the measuring day should be excluded from analysis. To establish inclusion and exclusion criteria differences of wear time between diary and NHANES algorithm were calculated in minutes for the entire cohort (n = 328) and for all measuring days (2,224 days). Based on the 10^th^ and 90^th^ percentile of the distribution the limits for exclusion were set to a difference of minus 45 and plus 150 minutes. This procedure follows the recommendation to accept a specified uncertainty in the PA data [18]. The negative value indicates that the sensor was worn according to the activity counts of the algorithm, although according to diary information the sensor has not been worn (e.g. NWT-sport). Conversely, a positive value indicates that the monitor was not taken off according to diary, whereas the NHANES algorithm suggests that the monitor was not worn due to low activity counts. Setting the evaluated limits for the discrepancies, 199 recording days exceeding the limits were excluded from analysis. Days with smaller discrepancies were included in the analysis using the diary entries for either wear time or NWT.

#### Visual inspection of PA

Activity count data were visually inspected for outliers or artefacts during the process of data download. After that, generated diagrams of PA data and comment based aspects from the diary were considered, as recommended [18]. The PA spent in each of the four intensity levels was displayed per day (Figure S3) and visually inspected for a first plausibility check. The aim was to gain a first impression of the individuals PA, i.e. to obtain information about its variability or regularity, whether the participant is active or not, the duration of physical activity, sport activities without wearing the monitor, and potential criteria of invalid days related to diary comments.

#### Handling of non-wear time during sport activities

Information about time and reason for not wearing the monitor was available through the diary entry. Monitors were not worn during certain sport activities, like soccer, martial arts, or swimming. Not taking into account these periods would lead to an underestimation of PA and misclassification of participants as being insufficiently active, especially, when sports were performed several times a week (e.g. swimming). Based on the information about kind of sports performed during NWT from the diary, the stated period of NWT-sport data was imputed according to the METs of the respective activity derived from the ‘Compendium of Energy Expenditures for Youth’ [34]. All sports with METs between 3 and 6 were assigned 100% to moderate intensity. In sports equivalent to 6 METs or higher the time was half assigned to moderate activity and half to vigorous activity. To provide a realistic representation of the time being active, 15 minutes were subtracted from the total NWT before data imputation to consider time for showering or transportation, which typically might be included in the stated time period. All days containing more than two hours of NWT-sport, for example a day of skiing or participating in sport contests, were automatically excluded from analysis (105 days) to avoid a false classification of minutes spent in moderate or vigorous activity.

#### Technical issues: identification of recording error

To gain information about biologically implausible data, activity counts were screened for extremely high values [28] and visually checked by the technical assistant. Three days were excluded from data analysis due to extremely high values caused by device errors.

#### Diary based data cleaning

**Figure 3:**
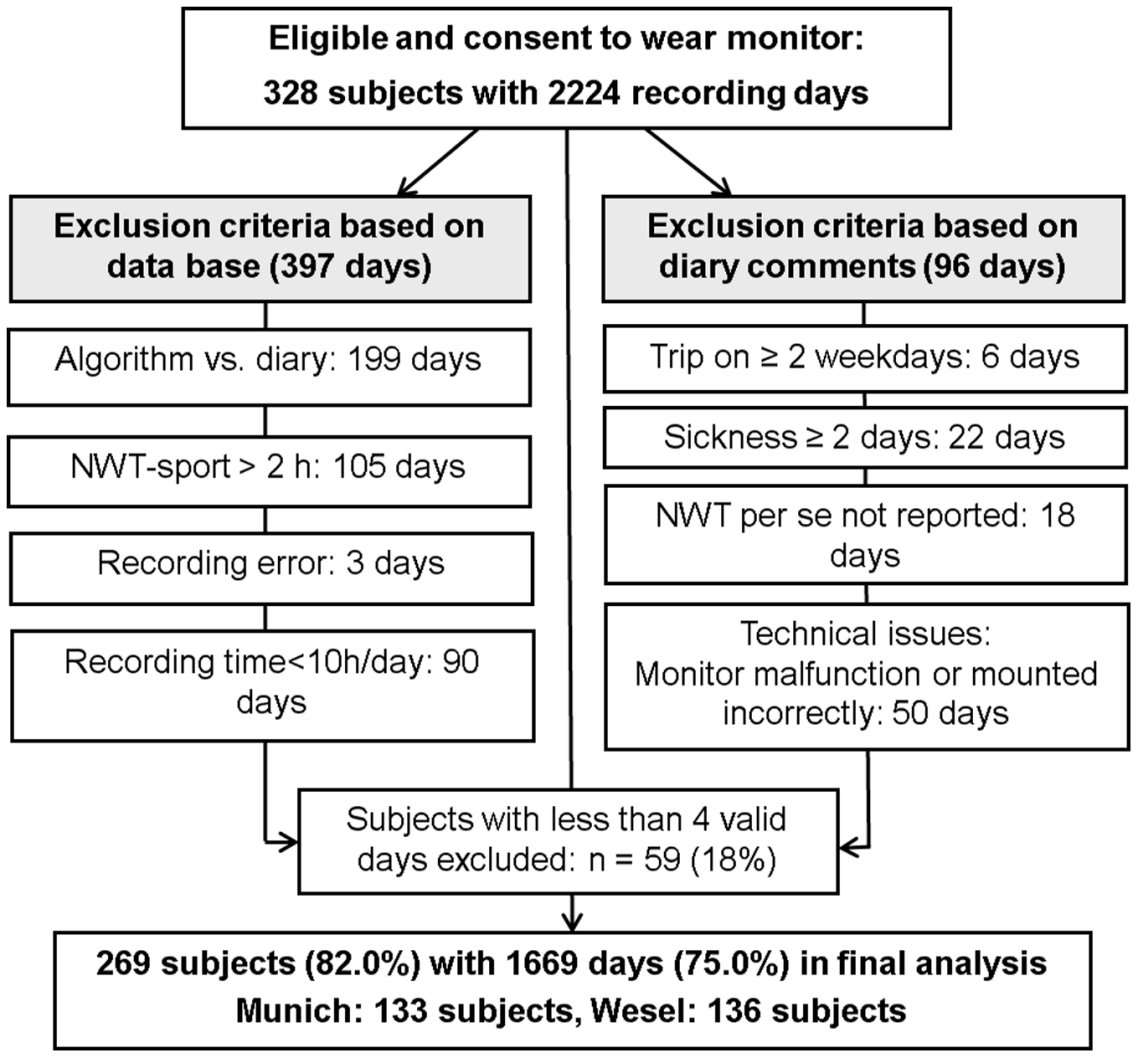
Exclusion of recorded days due to quality control in the ongoing GINIplus study. (NWT: non-wear time, NWT-sport: non-wear time during sport activities, algorithm: NHANES algorithm for detecting NWT [33]).

Specific events throughout the 7 days measuring period were considered as not being representative and, thus, the days were excluded from the analysis. Days with a diary reported common cold or headache were included in the analysis as was one day sick with staying in bed. But two or more days sick were excluded from the second day on. Accordingly, one day trips were included, whereas two and more days on a trip during the week, for example due to a school excursion, were excluded from the analysis. In case that for all 7 measuring days no information about NWT was provided by the participant, days were excluded from analysis (18 days). Sensor forgotten to wear was classified as NWT. In case the sensor was worn incorrectly (upside down or on hand instead of hip), or a flashing LED was reported suggesting technical issues, the corresponding days were excluded from the analysis (50 days, Figure 3).

#### Inclusion criteria

After quality control participants were included in the final analysis if they had recorded at least four complete days during the school week. At least one of these days had to be a weekend day. A day was considered valid if activity was recorded for at least 10 hours [18], [28].

### Statistical Analyses

All statistical analyses were conducted by using the R Statistical Programming Language for Windows, version 2.15.2 [35]. As main outcomes the mean minutes and their standard deviations spent in each of the four activity levels, and those spent in MVPA per day were calculated. Furthermore, the proportion of adolescents meeting the WHO recommendations of 60 minutes MVPA per day was determined. Participant characteristics were described using means and their standard deviations or proportions. Sex differences were analysed using chi square test for binominal variables. Wilcoxon Rank Sum Test was applied to compare continuous variables with two groups that were not normally distributed. When comparing more than two groups that were not normally distributed, Kruskal-Wallis Rank Sum Test was applied to test for global significance. Furthermore, pairwise Wilcoxon Rank Sum Test was applied to calculate pairwise comparisons between groups. The test corrects for multiple testing. Statistically significant differences were assumed at a significance level of p<0.05.

## Results

### Data Management and Quality Control

A total of 328 participants providing 2,224 recording days were subjected to the quality control criteria as described (Figure 2). After quality control, a total of 397 days (17.9% of recorded days) were considered invalid based on data base (Figure 3, left-hand side). In 50% (199 days) the discrepancy between wear time according to the NHANES algorithm [33] and diary data exceeded the accepted criteria of minus 45 minutes and plus 150 minutes per day. NWT-sport was reported in 144 cases. On 105 days the NWT-sport exceeded two hours, which is why these days were not included in the analysis. In isolated cases recording errors occurred. This led to an exclusion of 3 days. Furthermore, 90 days were below the minimum recording time of 10 hours a day.

Measured days were excluded on diary comments if the recorded activity was considered not to represent the typical routine over the week and, thus, every day routine of subjects appears to be violated (Figure 3, right-hand side). Six days were excluded because a trip was reported which lasts for more than two weekdays. 22 days were excluded because participants reported to feel sick on more than two days. On 50 days the devices were not worn correctly (e.g. worn on hand instead of hip or upside down, or a flashing LED had indicated technical issues). Out of the 328 subjects studied, three did not fill in the diary at all and were therefore excluded from the analysis (18 days).

Overall, 493 days (22.2%) of the 2,224 days were excluded from PA analysis after the quality check (Figure 3). Finally, of the 328 subjects 59 (18.0%) did not meet the minimum inclusion criteria of recording PA on at least three weekdays and one weekend day for 10 hours a day whereas 269 subjects (82.0%) were included in the final analysis providing a total of 1,669 recording days (75.0%).

### Sample Description

The final sample was comprised of 269 adolescents, 114 boys and 155 girls (42.4% vs. 57.6%, Table 1). The ratio of the regional distribution was almost 1∶1 (133 Munich, 136 Wesel). The mean age in both sexes was 15.5 years (±0.3 years, mean ± SD). The age ranges between 15.0 and 16.3 years. Boys were significantly taller (5.8%) and heavier (11.7%) than girls (p<0.001). However, the BMI (kg/cm^2^) did not significantly differ between sexes. On average, 84.4% of adolescents were considered normal weight defined as BMI ≥10 to <90 percentile [36], with a lower proportion of boys compared to girls (80.7% vs. 87.1%). Hence, the proportion of underweight (BMI <10 percentile, 10.5% vs. 6.5%) and overweight or obesity (BMI ≥90 percentile, 8.8% vs. 6.5%) was somewhat higher in boys compared to girls.

**Table 1:** Descriptive characteristics of adolescents.

Descriptive characteristics	Total sample	Boys	Girls
	(N = 269, 100%)	(N = 114, 42%)	(N = 155, 58%)
Region, n, Munich	133	63	70
Wesel	136	51	85
Mean (± SD)			
Age (y)	15.5 (0.3)	15.5 (0.3)	15.5 (0.3)
Height (cm)	172.0 (8.0)	177.7 (6.9)	167.9 (6.0)*
Weight (kg)	60.6 (10.1)	64.9 (11.0)	58.1 (8.5)*
BMI (kg/m^2^)	20.4 (2.6)	20.2 (2.6)	20.6 (2.6)
Weight status % (n/N)			
Normal weight	84.4 (227/269)	80.7 (92/114)	87.1 (135/155)
Underweight	8.2 (22/269)	10.5 (12/114)	6.5 (10/155)
Overweight and obese	7.4 (20/269)	8.8 (10/114)	6.5 (10/155)

* Significant differences between boys and girls, mean (± SD), p<0.001.

### Physical Activity

Eighty percent of the adolescents provided at least six valid measuring days. On average, girls and boys had an equal recording time of 14.8 h per day (Table 2). Overall, the minutes spent in each intensity level decreased with increasing intensity of PA. Two third of the 14.8 hours average daily wear time (incl. NWT-sport), i.e. about 10 hours, were spent in sedentary behaviour. The rest of wear time was mainly related to light activities (about 4.2 hours). Less than 5% of the time was spent in MVPA (4.4%): on average, 27.7 minutes (±16.4) in moderate, 11.4 minutes (±10.2) in vigorous intensity. In sum the mean minutes of MVPA per day were 39.1 minutes (±25.0).

**Table 2:** Mean (SD), % and 95% CI of PA intensity levels (minutes/day).

	Total sample	Boys	Girls
	(N = 269, 100%)	(N = 114, 42%)	(N = 155, 58%)
Total wear time	888.3 (41.9)	889.2 (101.6)	887.6 (42.8)
(incl. NWT-sport)			
[95% CI]	[883.6; 893.4]	[880.5; 895.4]	[881.2; 894.4]
Sedentary	598.0 (75.2), 67.3%	587.1 (75.0), 66.0%	605.9 (74.6), 68.2%*
[95% CI]	[592.5; 603.5]	[579.4; 596.1]	[598.4; 613.1]
Light	251.2 (54.6), 28.3%	260.3 (59.1), 29.3%	244.6 (50.3), 27.6%*
[95% CI]	[247.8; 255.2]	[254.2; 266.1]	[240.4; 249.6]
Moderate	27.7 (16.4), 3.1%	29.7 (14.3), 3.3%	26.2 (18.0), 3.0%*
[95% CI]	[26.4; 28.8]	[27.9; 31.3]	[24.6; 27.8]
Vigorous	11.4 (10.2), 1.3%	12.1 (9.7), 1.4%	10.9 (11.1), 1.2%
[95% CI]	[10.7; 12.2]	[10.9; 13.3]	[9.9; 11.9]
MVPA	39.1 (25.0), 4.4%	41.8 (21.5), 4.7%	37.1 (27.8), 4.2%*
[95% CI]	[37.7; 41.3]	[39.1; 44.3]	[34.8; 39.6]
Days with MVPA	20.6 (343/1669)	24.9 (179/718)	17.2 (164/951)
>60 min, % (n/N)			

* Significant differences between boys and girls, *p<0.001, NWT-sport: non-wear time during sport activities with imputed data due to diary comments on type of sport, MVPA = moderate-to-vigorous activity.

Girls spent 18.8 minutes more of the wear time in sedentary behaviour than boys (p<0.001), and showed less engagement in the other three intensity levels than boys. These differences were significant for light intensity (−15.7 minutes, p<0.001) and for moderate intensity activities (–3.5 minutes, p<0.001), whereas times (incl. NWT-sport) spent in vigorous activity were comparable between sexes: 12.1±9.7 minutes in boys vs. 10.9±11.1 min in girls. The cumulated duration of MVPA was significantly higher in boys compared to girls: 41.8±21.5 minutes vs. 37.1±27.8 minutes (p<0.001). The difference between boys and girls was on average 4.7 minutes, which corresponds to 12.7% of daily engagement in MVPA in girls.

### Proportion of Girls and Boys Meeting Daily 60 Minutes of MVPA

**Figure 4:**
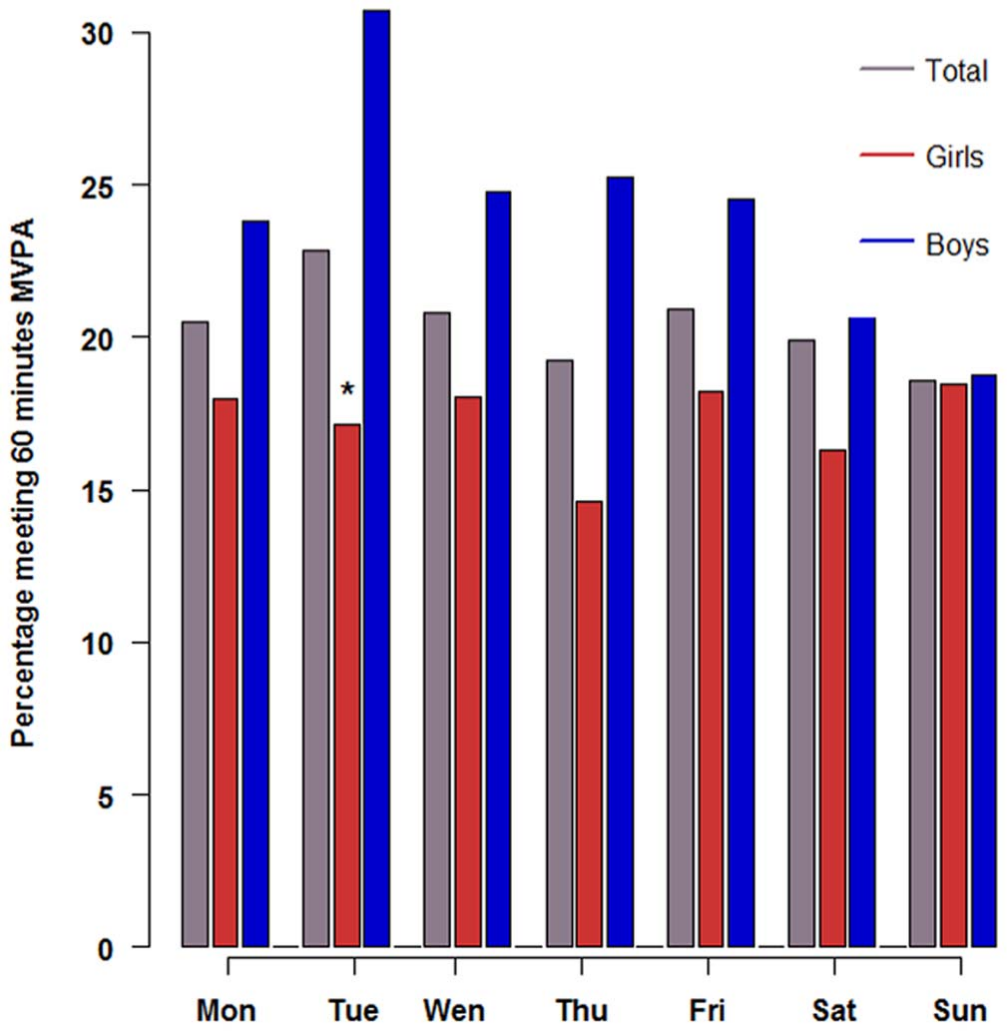
Percentage of girls and boys with at least 60 minutes of MVPA according to weekday (Mon-Sun). Differences between days were not significant in boys and of borderline significance in girls (p<0.054). *Significant differences between boys and girls, p = 0.02.

The proportions of adolescents who reach 60 minutes of MVPA a day was analysed according to sex and weekday (Figure 4). In the total sample the proportion of those meeting the WHO recommendations was evenly distributed throughout the week and ranged between 18.6% and 23.2% (ns). The sex stratified analysis showed that adolescents being compliant with the recommendations was significantly higher in boys compared to girls, on average 24.9% vs. 17.2% (p = 0.0003, Table 2). Significant differences between days were not detectable in boys although the Figure suggests that the proportion of those being compliant with the WHO recommendation was about 25% on all weekdays, except on Tuesday (30.7%), and somewhat lower during the weekend (19%, p = 0.53). Over the course of the week, a lower but more constant proportion of girls were engaged in at least 60 minutes of MVPA per day. The proportion ranged between 14.6% and 18.5%. The day with the lowest proportion of active girls was on Thursdays (14.6%, p = 0.054).

### Contribution of School Sport to Overall PA

Using information on PA intensity from the accelerometer and the available start and end times of school-based PA from the diary, the duration and proportion of MVPA that occurred during school sport was calculated. According to diary, the duration of the sport lesson was on average 79.9 minutes (±23.2) per week. Adolescents spent on average 17.2 minutes (±13.5) in MVPA during these lessons. The intensity of school-based MVPA differs substantially between individuals. Some adolescents engaged in MVPA for one third, whereas others engage in only 9% of total duration of the sport lesson. The proportion of school sports on total weekly MVPA was 6%, indicating that the contribution of school sport to total time spent in MVPA is quite low in adolescents.

### Type of Sport Performed during Leisure Time

The type of sport adolescents performed in their leisure time was of further interest. Some adolescents did either not specify the kind of sport, or did not give any information about the kind of sports at all (n = 36, 9.2%). A number of adolescents (n = 46, 17.1%) were engaged in at least two different sport activities so that in total 228 sport activities have been reported. All comments about kind of sports were categorized according to content (Table 3). Most adolescents engage in team ball sports (25.4%), such as soccer, handball and basketball. Endurance sports such as fitness or athletics were performed in 19.7% of participants. 16.2% participate in dancing class or gymnastics. Others engaged in individual ball sports such as tennis, badminton or table tennis (11.0%). Martial arts, winter sports, water sports and shooting sport each account for less than 5%. Other kinds of sports, which did not fit any category, were horse riding, cycling, roller-blading, weight lifting and climbing; these accounted for 16.7%. Boys predominantly engaged in ball sports (individual and team), whereas girls performed dancing and gymnastics and endurance sports.

**Table 3:** Kind of sports performed as reported by 228 adolescents, % (n/N).

Category	Total sample	Boys	Girls
	(N = 228)	(N = 93)	(N = 135)
Ball sports (team)	25.4% (58/228)	38.7% (36/93)	16.3% (22/135)
Endurance sports	19.7% (45/228)	18.3% (17/93)	20.7% (28/135)
Dancing and Gymnastics	16.2% (37/228)	6.5% (6/93)	23.0% (31/135)
Ball sports (individual)	11.0% (25/228)	15.1% (14/93)	8.1% (11/135)
Martial arts	3.9% (9/228)	3.2% (3/93)	4.4% (6/135)
Winter sports	3.9% (9/228)	6.5% (6/93)	2.2% (3/135)
Water sports	3.1% (7/228)	3.2% (3/93)	3.0% (4/135)
Other sports	16.7% (38/228)	8.4% (8/93)	22.2% (30/135)

## Discussion

Assessment of PA by accelerometry in large scale studies results in a huge amount of data which have to be subjected to standardised data management before the final PA analysis can be conducted. Up to now, recommendations related to different aspects of PA measurement exists [18], [20], [28], however, there is a need for a more comprehensive approach to standardize the process of data management [18]. Here we introduce an approach for the assessment of PA in adolescents which combines accelerometry and diary based information and provides standards for identification of valid data and data reduction procedures. Based on the data obtained in more than 300 German adolescents, major aspects of data management have been addressed, such as validity of wear time, handling of NWT, and inclusion of diary comments. Diary information was shown to provide important information for interpretation of accelerometer data and for gaining additional information about PA in adolescents so that it appears to be a need for solid interpretation of accelerometry data. Our approach identifies a reasonable number of valid days and compliant participants so that we suggest the use for management of PA data recorded in adolescents. First results of objectively measured PA in German adolescents suggest that four out of five German adolescents do not meet the WHO recommendations for PA.

### Data Management and Quality Control

A major concern is that NWT often is related to non-compliance of study participants, a considerable problem in adolescents [28]. Often consecutive minutes of zero-output are identified as NWT and not considered for data analysis, which is likely to result in a data bias [21]. On the other hand, NWT due to legitimately removing the monitor during aquatic activities or for safety reasons may result in an underestimation of PA [20]. To overcome this problem, non-wear time activity diaries have been recommended [20], [37]. However, the application of diaries in adolescents is associated with some uncertainty, especially with regard to NWT [38]. Thus, our approach used diary comments and objective criteria derived from the activity counts to validate wear-time and identify periods of NWT. NWTs identified by the NHANES algorithm [33] were compared with the corresponding times reported in the diary. Overall, there was good agreement, but also discrepancies spanning some minutes up to a maximum of an entire day. Since not only the diary information of the adolescents may be invalid, but also the algorithm may misclassify NWT [39], we propose cut off limits for exclusion of invalid information based on our data, i.e. differences between algorithm and diary entry exceeding minus 45 minutes and plus 150 minutes were not accepted. Setting these limits, we accept a specified uncertainty at the advantage of an increased sample size, as recommended recently [18]. Using our limits, a reasonable number of days (8.9% of recorded days) were excluded but 35.8% with differences below the limit kept for further analysis. The potential inaccuracy of information given about NWTs in the diary appears to be confirmed in our study, as the highest number of days excluded was due to discrepancies of wear-time between diary and algorithm. However, in most cases the provided diary information was acceptable. In this sense, the diary appears to be a useful tool and additional information about NWT during sport activities (NWT-sport) can be used for data imputation. For NWT-sport up to 2 hours, moderate or vigorous activities were imputed based on the METs [34] corresponding to the type of sport performed, as suggested recently [20], [21]. We used a cut off limit of 2 hours for NWT-sport to avoid a false classification of the minutes spent in moderate or vigorous activity. Typically, NWT-sport >2 hours lasts for longer periods and was related to daily activities, like skiing for a day or participation in sport contents. We feel that these data could not be reasonably imputed because they are likely to include transportation and breaks and intermittent phases of exercise which are hard to assess. NWT per se was not reported in less than 1% of the recorded days indicating that this is of minor concern in adolescents. Thus, a major fraction of recorded days was excluded due to uncertainties related to NWT which is comparable to recently published data [20]. Of minor concern in the present study were technical issues and days considered not to represent every day routine of subjects. A minimum daily wear time of 10 hours was accepted in line with results from Colley et al. [28] in adolescents commonly applied in the research community. Exclusion of only 4% of recorded days suggests that this limit adequately balances between a selection bias due to too stringent limits of 12 hours or 14 hours a day and the representativity of the included days for the subjects’ every day routine. After exclusion of invalid days, subjects with less than 4 valid days including at least 1 weekend day were excluded from the analysis. This was true for 18% of the participants, a little bit lower than the percentage reported by Colley et al. [28] for adolescents (23%), but similar to that reported for HELENA [17]. The percentage of participants with at least 6 valid days of accelerometer wear was 65% in the present study, which exceeds the 53% reported by Colley et al. [28] and the 56% observed in HELENA [17], but is similar to the 63% reported by Ottevaere et al. [20]. Thus, our approach for data cleaning considering several recently published recommendations [18], [20], [21], [28] provides reasonable data with regard to the number of days and participants included for final data analysis.

### Physical Activity in German Adolescents

The present study provides the first data on objectively measured PA in German adolescents. The total wear time did not significantly differ between girls and boys, but was almost 2 hours longer than that reported for the HELENA study in 12.5 to 17.5 years old adolescents [17]. The commonly observed sex difference for PA was reproduced in our study. Consistent with findings reported in HELENA [17], a higher engagement in sedentary behaviour was found for girls, whereas boys engaged in more minutes of MVPA. However, estimated minutes in MVPA were 40% higher in the HELENA study sample, with a difference of 15.9 minutes in mean daily MVPA (55 minutes vs. 39.1 minutes). Differences were more pronounced in boys (22.2 minutes, 53%) than in girls (11.9 minutes, 32%). Even after correction for the lower threshold applied to define moderate intensity in the HELENA study (2000 cpm in HELENA vs. 2200 cpm in GINIplus), which accounts for about 4 minutes in MVPA [40], the engagement in MVPA was still higher in the HELENA study sample. Also the percentage of participants who met the WHO recommendation of PA was considerable lower in our study: 41% in HELENA vs. 21% in GINIplus. The difference was more pronounced for boys than for girls, the ratio being 2.3 for boys and 1.6 for girls. Analysis of the proportion meeting the 60 minutes MVPA according to weekday gave no clear pattern in both sexes, but boys appear to be more active during the weekdays than on the weekend, whereas girls exhibit a more constant activity throughout the week. Although our results are preliminary, they suggest that German adolescents are less active than the average from different European cities represented by the HELENA study. Beside methodological aspects, various reasons may account for this, such as social, cultural, or environmental factors known to differ among European countries.

The percentage of 15-year-old German adolescents who met the WHO recommendations of PA has been assessed by questionnaire in the HBSC and KIGGS study [13], [14]. Whereas, compared to our results, lower values were reported in the HBSC study for boys (13% vs. 25% in GINIplus) and girls (9% vs. 17% in GINIplus), nearly the same values were obtained in the KIGGS study, 25% and 17%, respectively. These data indicate that four out of five adolescents from Germany do not meet the WHO criteria on PA, despite the fact that we applied in our preliminary analysis the “Child×Day” approach, which is considered to overestimate MVPA compared to the “All Days” method [41].

The question to what extent school sport contributes to total weekly activity of adolescents is an example of specific aspects we attempt to address with our accelerometer and diary based approach. Our results show that school sport contributes very little to total time spent in MVPA, on average only 6%. Moreover, only 22% of the school sport time was spent in MVPA. Therefore, school sport does not have a high impact on adolescents’ engagement in MVPA. Instead, sports in leisure time appear to account for weekly activity and should therefore be promoted in interventional studies. Most of the adolescents are engaged in team and endurance sports, as well as in dancing and gymnastics, which is comparable to results recently reported for adolescents from Belgium, although swimming was found to be much lower in GINIplus adolescents (<5% vs. 25%, [20], [21]).

### Study Limitations

First of all, we have to emphasize that the data on PA are inferred from a subsample of the ongoing GINIplus study. However, due to the scarcity of accelerometry data from German adolescents we felt that, besides introducing the data management for GINIplus, preliminary results from a considerable study sample of finally 269 subjects are of interest for the research community. The sample comprised of adolescents from Munich and Wesel, recruited as follow-up of the birth cohort. Therefore, the sample is likely to have a certain selection bias and might not be representative for German adolescents. Further, a selection bias in terms of recruitment must be assumed, due to voluntary participation. Those adolescents included in the study sample might be more interested in PA or engage in sufficient minutes of MVPA, which is why they agree to conduct the accelerometry. The low proportion of overweight and obese adolescents included in our study sample support this assumption. As a result, an overestimation of the mean minutes spent in MVPA is to be expected, because those of higher weight are considered to be less active [11]. In this regard, the high proportion of adolescents who denied participation after receiving the monitors (29.0%), has to be mentioned. Although we did not study this aspect in a standardized manner, reasons provided by the adolescents in the diary or after a phone call by the technician were mainly that adolescents feel uncomfortable to wear the monitor after tying it, due to its size or appearance. Particularly girls often argued that monitors are not so easy to hide under the clothing. In only few cases, disqualifying comments from friends or school mates such as ‘being under electronic monitoring’ were mentioned. Also incompliance related to pubertal behaviour became apparent during the phone call. In addition, the fact that we do not distribute the device face-to-face, and the incentive to receive a result letter of their own PA, may be inadequate to attract adolescents to wear the monitor [18]. In summary, the acceptance to wear the device was less than three quarters. This is important when representativity and generalisability of the data are discussed.

Although adolescents were instructed to follow their daily routine, there might be a bias towards higher activity because of being “observed” by wearing the monitor. This bias is expected to be highest on the first day of recording [42]. The majority of adolescents started measuring on a Monday, followed by Friday and Thursday (40.2%, 16.4%, 15.2%, respectively). Comparing the minutes of MVPA on each weekday, MVPA was not significantly higher on the first measuring day, suggesting that “observation bias” is of minor importance in the present study. Furthermore, there might be a selection bias due to the process of data cleaning. However, current recommendations for data management [18], [20], [28] have been applied, and the percentage of invalid days and subjects to be excluded from analysis were comparable to other studies in adolescents.

### Conclusions

We introduced an accelerometer and diary based approach for assessment of PA in adolescents combining several aspects of data management recently recommended. Applied in 15-year old adolescents, a reasonable number of invalid days and incompliant participants have been identified and diary based information was used to gain additional information about PA in adolescents. It is therefore suggested to consider this approach for management of PA data recorded in adolescents. Although the results on PA are preliminary and have to be valued with precaution, our accelerometer based results on PA in German adolescents were in the range of those from the KIGGS and the HBSC study, even though these studies were questionnaire-based. Our study suggests that four out of five adolescents from Germany do not meet the WHO criteria on PA, and that German adolescents are likely to be less active than the European average introduced by the HELENA study.

## Supporting Information

Figure S1 **Result letter for participants.** The result letter is thanking the adolescent for participating in the study and is introducing the different levels of physical activity in a common sense. Two diagrams provide the relative time the adolescent has spent in the different levels of activity separately for weekdays and for the weekend. The 60 minutes of MVP recommended by the WHO are introduced and the mean minutes, the minimum minutes and maximum minutes the adolescents spent in MVPA per day are given.
(DOCX)

Figure S2 **Activity diary for seven days.** In the head, ID, anthropometric information, participant’s handedness and hand side of wearing the monitor is documented. For recording the course of day a detailed schema is provided for each day covering from the morning on corner stones of the day. Further, options are provided for time and reason of removing monitors (non-wear time) and for remarks. A column labelled in blue illustrates in a sample how to fill in the diary.
(DOCX)

Figure S3 **Graphical illustration of wear time according to diary and NHANES algorithm (left) and time of PA spend in each of the four intensity levels (right) demonstrated in four samples.** Left: Graphical illustration of the wear time according to the NHANES algorithm [33] and diary information as a function of time of the day and number of recorded day 1 to 7. Examples cover an almost perfect matching of wear time between diary and algorithm (Participant A) to obvious discrepancies between algorithm and diary (Participant C). Right: The minutes of PA spent in each of the four intensity levels are displayed per day based on diary information before data cleaning. Little engagement in MVPA (Participant C), typical levels of MVPA throughout the week (Participant A), and regularly engagement in MVPA (Participant B) are shown.
(DOCX)
