# Supplementary material for: Physical Activity in German Adolescents Measured by Accelerometry and Activity Diary: Introducing a Comprehensive Approach for Data Management and Preliminary Results
Source: PLoS One. 2013 Jun 4;8(6):e65192. doi: 10.1371/journal.pone.0065192 (PMC3672153; doi:10.1371/journal.pone.0065192)
Supplement: Figure S1 — Result letter for participants. The result letter is thanking the adolescent for participating in the study and is introducing the different levels of physical activity in a common sense. Two diagrams provide the relative time the adolescent has spent in the different levels of activity separately for weekdays and for the weekend. The 60 minutes of MVP recommended by the WHO are introduced and the mean minutes, the minimum minutes and maximum minutes the adolescents spent in MVPA per day are given. (DOCX) [file pone.0065192.s001.docx]

**Figure S1. Result letter for participants.**


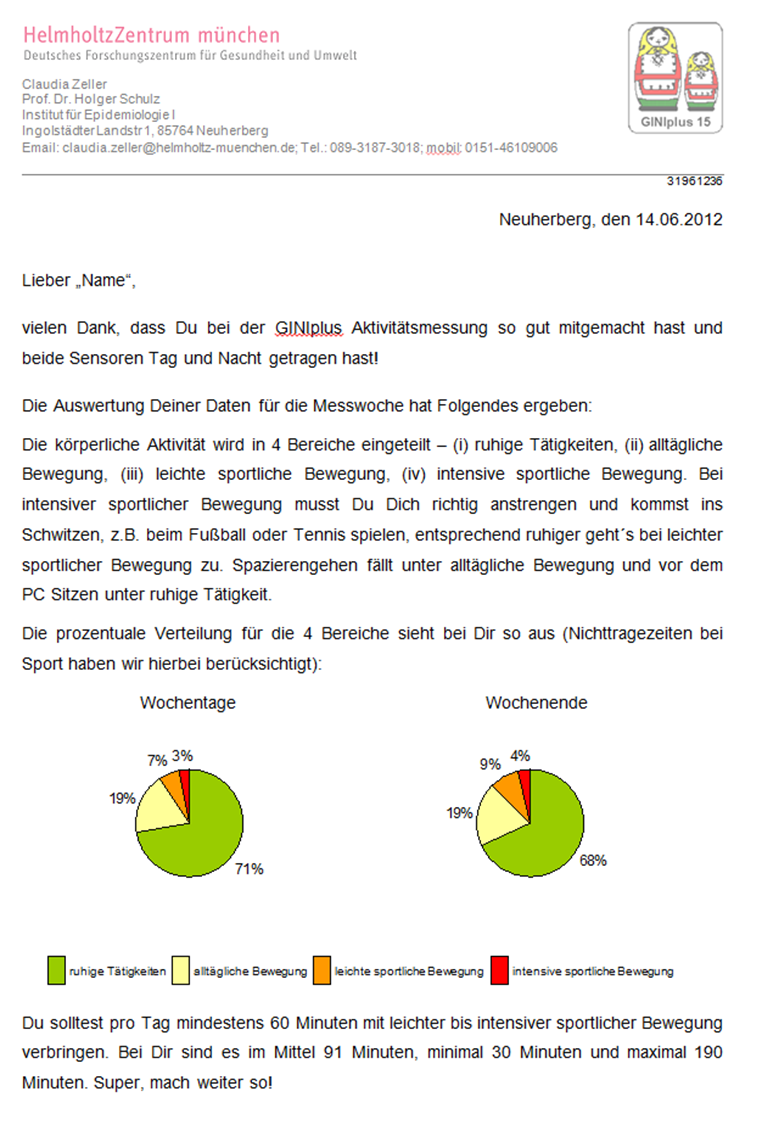


The result letter is thanking the adolescent for participating in the study and is introducing the different levels of physical activity in a common sense. Two diagrams provide the relative time the adolescent has spent in sedentary (green), light (yellow), moderate (orange) and vigorous activity (red) separately for weekdays (left) and for the weekend (right). The 60 minutes of MVP recommended by the WHO are introduced and the mean minutes, the minimum minutes and maximum minutes the adolescents spent in MVPA per day are given. Finally, in this case, the adolescent is encouraged to keep his high PA level.
