# Supplementary material for: Physical Activity in German Adolescents Measured by Accelerometry and Activity Diary: Introducing a Comprehensive Approach for Data Management and Preliminary Results
Source: PLoS One. 2013 Jun 4;8(6):e65192. doi: 10.1371/journal.pone.0065192 (PMC3672153; doi:10.1371/journal.pone.0065192)
Supplement: Figure S2 — Activity diary for seven days. In the head, ID, anthropometric information, participant’s handedness and hand side of wearing the monitor is documented. For recording the course of day a detailed schema is provided for each day covering from the morning on corner stones of the day. Further, options are provided for time and reason of removing monitors (non-wear time) and for remarks. A column labelled in blue illustrates in a sample how to fill in the diary. (DOCX) [file pone.0065192.s002.docx]

**Figure S2. Activity diary for seven days**

**Diary for day 1 to 4**


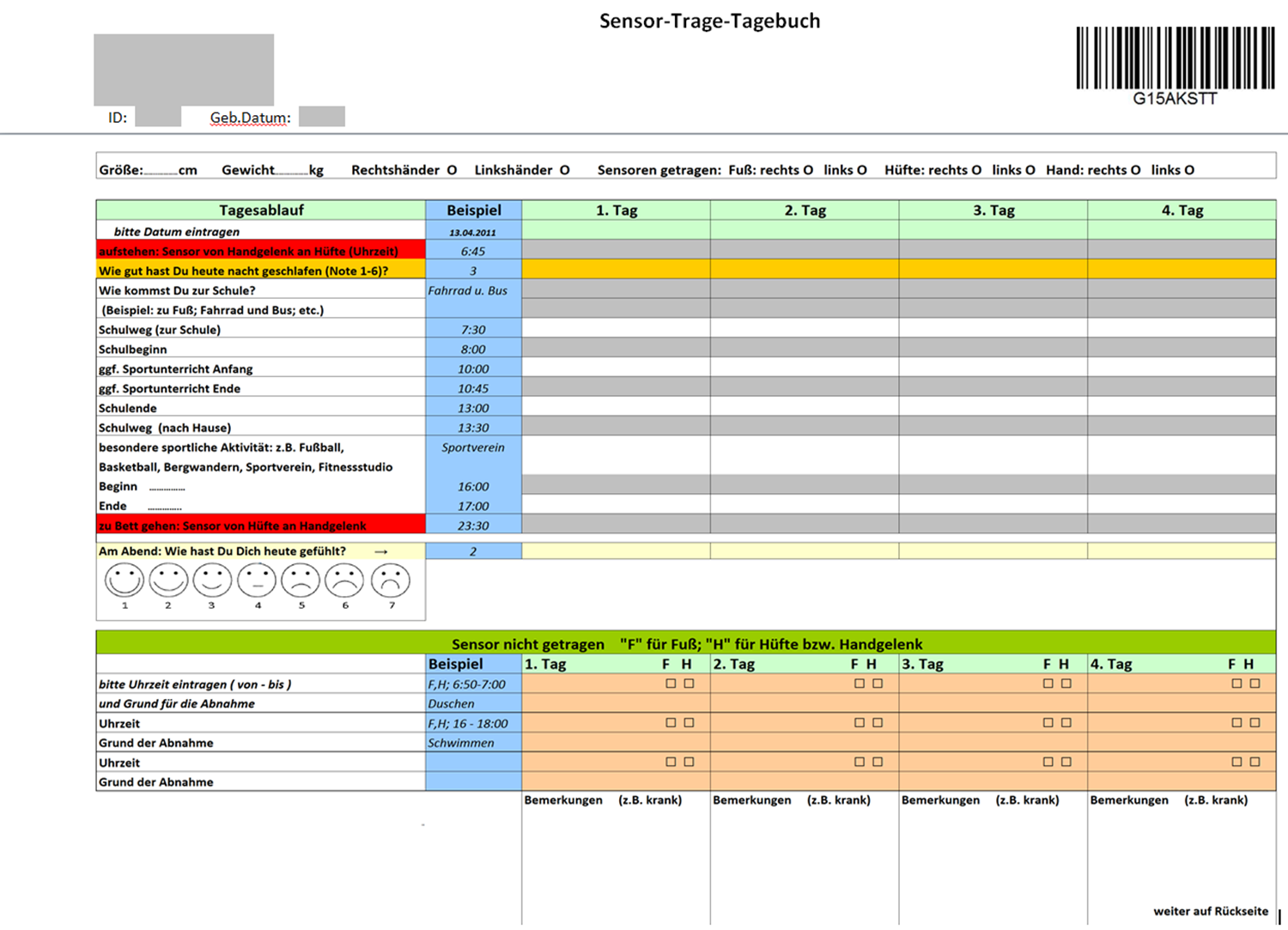


**Diary for day 5 to 7**


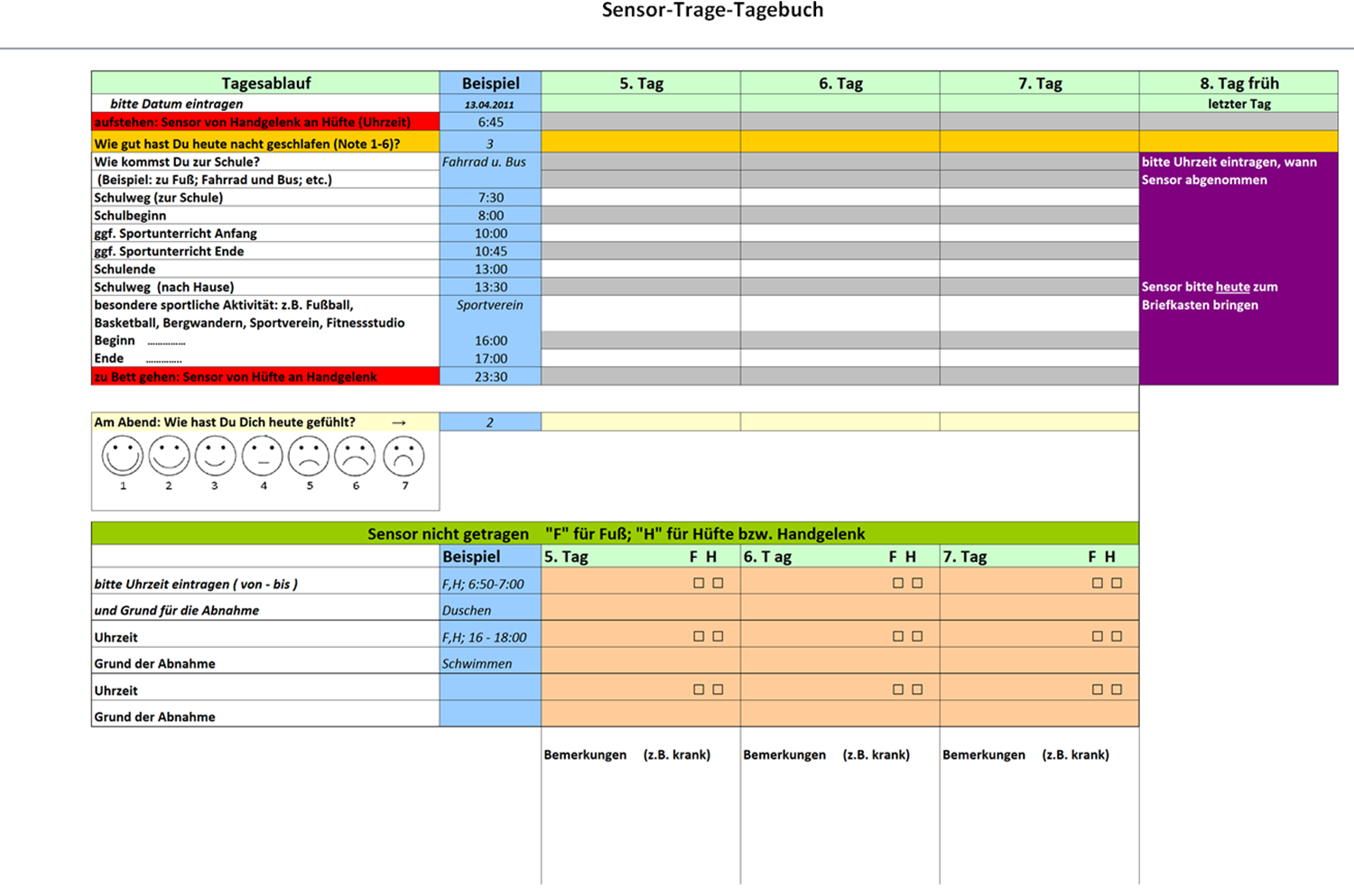


In the head, ID, anthropometric information such as weight and height, participant’s handedness and hand side of wearing the monitor is documented. For recording the course of day a detailed schema is provided for each day covering from the morning on the following items: date; getting up and changing the sensor from hand to hip; ranking how the adolescent slept the night; time of travel to school and kind of transportation; start of school; if any, start and end of school sport; end of school; time of travel from school to home and kind of transportation; time when reaching home; leisure sport activities: beginning, end, and type of activity; time of going to bed and changing sensor from hip to hand; ranking how the adolescent felt the day. Further below, options are provided for time and reason of removing one or both monitors (non-wear time) and for remarks. The column labelled in blue illustrates in a sample how to fill in the diary.
