# Supplementary material for: Physical Activity in German Adolescents Measured by Accelerometry and Activity Diary: Introducing a Comprehensive Approach for Data Management and Preliminary Results
Source: PLoS One. 2013 Jun 4;8(6):e65192. doi: 10.1371/journal.pone.0065192 (PMC3672153; doi:10.1371/journal.pone.0065192)
Supplement: Figure S3 — Graphical illustration of wear time according to diary and NHANES algorithm (left) and time of PA spend in each of the four intensity levels (right) demonstrated in four samples. Left: Graphical illustration of the wear time according to the NHANES algorithm [33] and diary information as a function of time of the day and number of recorded day 1 to 7. Examples cover an almost perfect matching of wear time between diary and algorithm (Participant A) to obvious discrepancies between algorithm and diary (Participant C). Right: The minutes of PA spent in each of the four intensity levels are displayed per day based on diary information before data cleaning. Little engagement in MVPA (Participant C), typical levels of MVPA throughout the week (Participant A), and regularly engagement in MVPA (Participant B) are shown. (DOCX) [file pone.0065192.s003.docx]

**Figure S3. Graphical illustration of wear time according to diary and NHANES algorithm (left) and time of PA spend in each of the four intensity levels (right) demonstrated in four samples.**


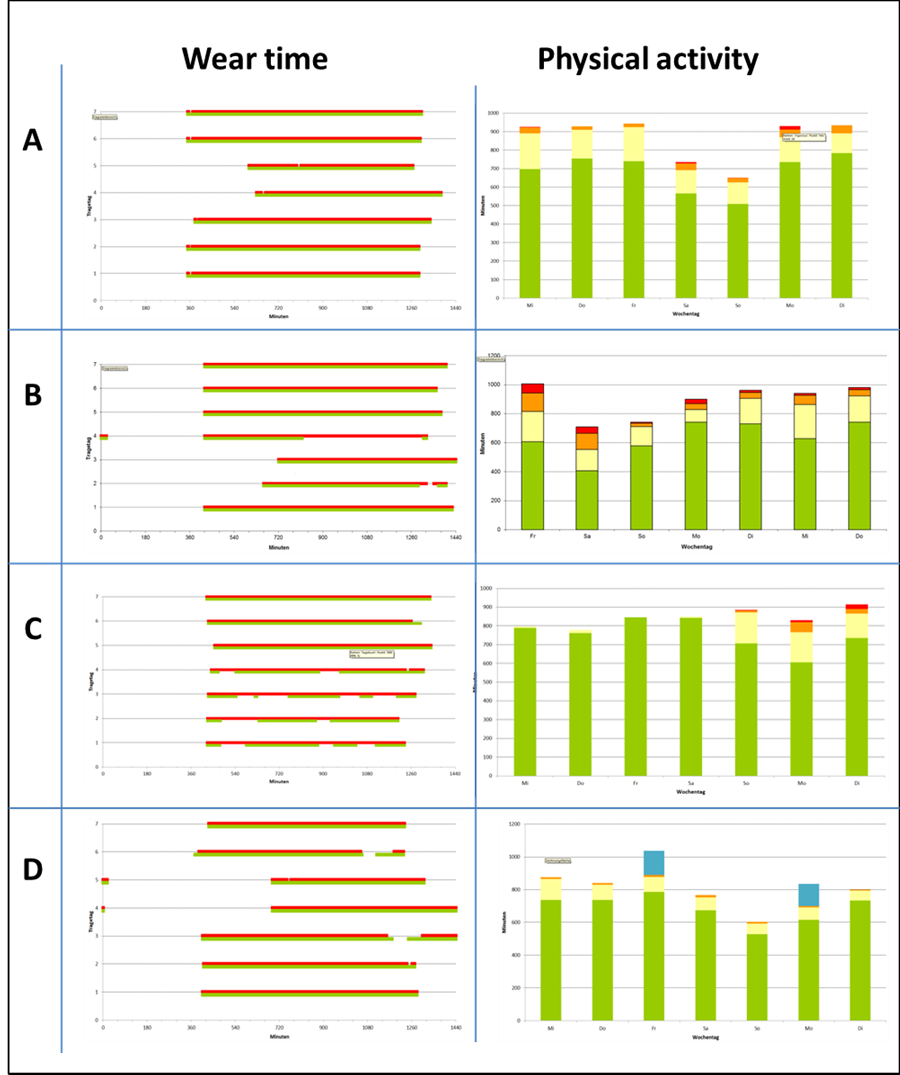


**Left:** Graphical illustration of the wear time according to the NHANES algorithm [[33](#_ENREF_33)], green, and diary information, red, as a function of time of the day (minutes starting from midnight on). Y-axis provides the recorded days 1 to 7 from bottom up.

**Right:** The minutes of PA spent in each of the four intensity levels are displayed per day based on diary information before data cleaning (sedentary in green; light in yellow; moderate in orange, vigorous in red, NWT-sport in blue).

**Participant A:** An almost perfect matching of wear time between diary and algorithm is visible and typical levels of MVPA throughout the week are displayed, day 4 and 5 represent Saturday and Sunday.

**Participant B:** Minor discrepancies of wear time are detectable on day 2 but a major one on day 4, which results in the exclusion of day 4. The participant is regularly engaged in MVPA.

**Participant C:** concerning the wear time, obvious discrepancies between algorithm and diary are visible for day 1 to day 4, resulting in the exclusion of days 2 and 3. The participant appears to be little engaged in MVPA.

**Participant D:** An almost perfect matching of wear time is given except for day 3 and 6 due to NWT-sport (martial arts). Physical activity throughout the week is almost exclusively achieved by NWT-sport on day 3 (Friday) and 6 (Monday).
